# An Ethnography Study of a Viral YouTube Educational Video in Ecuador: Dealing With Death and Grief in Times of COVID-19

**DOI:** 10.3389/fpsyt.2021.648569

**Published:** 2021-07-09

**Authors:** Lydia Giménez-Llort

**Affiliations:** ^1^Medical Psychology Unit, Department of Psychiatry and Forensic Medicine, School of Medicine, Universitat Autònoma de Barcelona, Barcelona, Spain; ^2^Institut de Neurociències, Universitat Autònoma de Barcelona, Barcelona, Spain

**Keywords:** pre-school education, death, grief, disenfranchised grief, youtube, ethnography, COVID-19

## Abstract

In Western societies, death is a social and educational taboo. Poor education about death and mourning processes and overprotective family and social attitudes move children away from death to avoid “unnecessary suffering.” The COVID-19 outbreak highlighted these shortcomings and the difficult management of grief's complexity under sudden and unexpected scenarios. The need for immediate and constant updates related to COVID-19 benefited from social media coverage's immediacy. The use of YouTube as a digital platform to disseminate/search for knowledge exploded, raising the need to conduct ethnographic studies to describe this community's people and culture and improve the booming social media's educational capacity and quality. The present virtual ethnography studied 255,862 YouTube views/users and their behavior related to “Vuela Mariposa, Vuela,” a children's story available online since 2009 (not monetized) about the cycle of life, death, and disenfranchised grief (not acknowledged by society) that went viral (+>999%) on May. To our knowledge, this case study is the first original research that explores the ethnography of (i) a viral video, (ii) on death and grief taboo topics, (iii) for prescholars, and (iv) before and during the COVID pandemic. The quantitative and qualitative analyses identified a change in the users' profiles, engagement, and feedback. During the previous 11 years, the users were 35–44 years old Mexican and Spanish women. Those in grief used narrative comments to explain their vital crisis and express their sorrow. In the pandemic, the analysis pointed to Ecuador as the virality geographical niche in a moment when the tragic scenarios in its streets were yet unknown. The timeline match with the official records confirmed the severity of their pandemic scenario. The viral video reached a broad population, with normal distribution for age, and including male gender. Engagement by non-subscribers, direct search (traffic sources), and mean visualization times suggested educational purposes as confirmed by the users' feedback with critical thinking referring to the cycle of life's meaning and societal mourning. For the youngest users, the video was part of academic assignments. The ethnography pointed at YouTube as a flexible education resource, immediately reaching diverse users, and being highly sensitive to critical events.

## Introduction

In many Western societies, focused on youth and productivity, after a century of health and social advances increasing life expectancy by half a century, death is a taboo (1–3). Most people prefer not to think about it unless it is necessary. They behave as if death will exclusively be the endpoint of old age, and the “word taboo” forces the use of euphemisms. Talking about death is considered inappropriate or offensive outside normative settings restricted to figurative scenarios, the religious doctrine, or when its use is unavoidable in medical situations. The taboo on the dead includes naming the deceased person, touching the dead, those encompassing them, and anything related to it. Grief that does not align with social norms, the so-called disenfranchised grief, is not acknowledged by society and even denied. In other societies, indigenous ceremonies and rituals like those of Mexicans or Australian aborigines provide the opposite scenario, where the celebration of death and dead people are intrinsic to their culture (4, 5).

The emerging, rapidly evolving nature of the COVID-19 scenario results in a strong need for fast and freely accessible medical information resources to spread recommendations, guidelines, and constantly updated advice (6). Internet was already a popular source of healthcare information for both patients and professionals through not only different “social media” formats, such as blogs, microblogs (Twitter), and forums in medical websites, but also social networking sites (i.e., LinkedIn and Facebook) and communities (YouTube) (General Medical Council UK (https://www.gmc-uk.org) (7)). In particular, YouTube is the second most popular website in the world, and its use as a digital platform to disseminate knowledge in the health field is on the rise, along with its ability to be a disinformation niche (8). The increased use of YouTube has highlighted the importance of conducting ethnographic studies to describe this community's culture. This research approach can be defined as studying social interactions, behaviors, and perceptions within groups and communities (9). It is characterized by exploring the nature of a particular social phenomenon rather than testing a hypothesis about it. This research model applied in virtual education aims to improve the educational capacity and quality of the booming social media (10).

Thanatology and current practice in death education are an emerging field in postmodern Western societies, using interdisciplinary approaches (11) to counteract the social taboo of death (12). Poor education about death and mourning processes, together with overprotective family and social attitudes, moves children away from death to avoid what is considered an “unnecessary suffering” (13, 14). The COVID-19 pandemic has highlighted these shortcomings (15, 16) and the severe difficulties that individuals, but mostly society, have to manage grief's complexity under adverse scenarios (17–19) and chronosystem pressure (20). The situation is worsened by multiple mourning, as to the loss of the loved ones, individuals add other material, economic, and social losses that increase the meaning and impact. In children, the protective attitudes postpone their “confrontation” with the concept of death, instead of letting it be a natural part of the child's psychology and maturational development. More recently, among health promotion perspectives, digital storytelling (21) and children's literature have been foreseen to promote students' global development and well-being (22). The approach of death and mourning process through tales is a common educational resource used since prescholar times (23–25) similarly to occasional “teachable moments” where children are in contact with the presence of finitude (26). Under a psychosocial nursing perspective, the most recent work also refers to the relevance of using storytelling with grief reactions in children during the COVID-19 pandemic (27). Experts consider that death education programs are a kind of intervention program to learn coping strategies to deal with the fear of death and manage loss and anxiety (28), which are important to incorporate also into school curricula (29).

The present work is presented as a case study aimed to determine the ethnographic profile of 255,862 YouTube users (as estimated from visualizations) and their behavior related to “Vuela Mariposa, Vuela,” a children's story available online since 2009 (not monetized) about the cycle of life, death, and disenfranchised grief (not acknowledged by society) that went viral (+>999%) on May 2020. To our knowledge, this brief report is the first original research that explores the ethnography describing the YouTube community's people and culture associated with (i) a viral video, (ii) dealing with death and grief taboo topics, (iii) created as a material for prescholars, and (iv) quantifying the change in the ethnography profile before and during the COVID-19 pandemic. The children's story was first published open access in Encuentos.com, a digital editorial before the illustrated book, which also includes a guide for parents and teachers, was published in an independent editorial. The story is also part of a scholarly book for American students. Its professional use is cited in a doctoral thesis and several professional websites and blogs of Spanish and Latino–American psychologists specialized in managing children's grief.

## Material

The material consists of two videos hosted on a non-monetized YouTube channel. They present a Spanish children's story on the cycle of life, death, and disenfranchised grief entitled “¡Vuela, mariposa! ¡Vuela!” (30) and its English version “Fly, Butterfly, Fly!” (31), both original works of the author of the present report. The original version was published on June 23, 2009, and in its English version 2 days later, on June 25 of the same year.

### Participants

This study's participants are constituted by an independent, anonymous sample of 255,862 YouTube users, an estimated number by YouTube Analytics based on the number of views from their publication dates (June 23 and 25, 2009) until August 31, 2020. In each ethnography analysis (Tables 1–6), the number of estimated users (views) is indicated. The sample of interest refers to the time frames where the virality of the video occurred. It is focused on the 96,019 YouTube views in the 174 days since the start of the official declaration of the pandemic (WHO, March 11, 2020) until August 31, 2020 (Tables 1–3). After that, the 23,246 users (views) on the day that went viral determine the viral video's engagement indicators (Table 4). Service used to share was determined from the behavior of 6,949 users, from whom 3,944 users belong to the historic pre-pandemic period and 3,005 users to the pandemic (Table 5). Finally, a total sample of 90 users interacted in the YouTube channel to write commentaries to the video, thus providing feedback during the pre-pandemic (33 users) and the pandemic (57 users) periods.

**Table 1:** Comparative analysis on the use of the children's story on cycle of life, death, and grief—indicators and traffic sources.

	**!Vuela Mariposa, Vuela!**		**Fly, butterfly, Fly!**	
	**(A) Historic**	**(B) Pandemic**	**(A) Historic**	**(B) Pandemic**
	**2009 to March 10, 2020**	**March 11 to August 31, 2020**	**2009 to March 10, 2020**	**March 11 to August 31, 2020**
**Indicator**	***n***	***n***	***n***	***n***
Visualizations (total: 255,592)	159,573	96,019	1,203	67
Impressions (total: 336,980)	218,892	118,088	398	243
Time (h) of visualization (total: 13,454)	6,662.3	6,791.7	8.1	0.7
Mean duration of the visualizations	3:01	4:14	1:39	1:27
Subscribers (total: 624)	200	424	0	0
Percentage of clicks (%)	5.6	10.0	1.0	1.2
**Traffic source**	***n*** **(%)**	***n*** **(%)**	***n*** **(%)**	***n*** **(%)**
External source	**55,220 (34.6)**	32,702 (34.1)	104 (8.2)	3 (4.5)
Direct or unknown	19,628 (12.3)	46,615 (48.6)^***^	**286 (23.8)**	26 (38.8)^*^
YouTube search	44,080 (27.6)	11,738 (12.2)	**286 (23.8)**	2 (3.0)
Suggested videos	20,399 (12.8)	2,312 (2.4)	175 (14.6)	33 (49.3)^***^
Inserted	7,085 (4.4)	—	94 (7.8)	—
Other YouTube functions	2,920 (1.8)	1,749 (1.8)	50 (4.2)	2 (3.0)
Google search	3,487 (2.2)	—	11 (0.9)	—
Promotion for YouTube partners	2,268 (1.4)	—	162 (13.5)	—

*Data for (A) Spanish version and (B) English version of the children's story are expressed as real numbers (n) and percentages (%). In bold, the highest percentages are indicated. The symbol “—” is meant for absence or data not reported or quantifiable, according to YouTube analytics. Statistics: χ^2^*,

* *p = 0.05*,

*** *p = 0.0001 vs. pre-pandemic period*.

**Table 2:** Comparative analysis on the use of the children's story on cycle of life, death, and grief—age and sex.

	**!Vuela Mariposa, Vuela!**		**Fly, butterfly, Fly!**	
	**(A) Historic**	**(B) Pandemic**	**(A) Historic**	**(B) Pandemic**
	**2009 to March 10, 2020**	**March 11 to August 31, 2020**	**2009 to March 10, 2020**	**March 11 to August 31, 2020**
**Age**	**% Users, mean duration of visualization and (%)**	**% Users, mean duration of visualization and (%)**	**%, Mean, %**	**%, Mean, %**
13–17 years old	—	4.1%, 3:46 (47.0)	—	—
18–24 years old	—	14.3%,^***^ 4:06 (51.1)	—	—
25–34 years old	—	**25.8%**,^***^ **4:13 (52.6)**	—	—
**35**–**44 years old**	100%, 2:51 (35.5)	**24.8%**,^***^ **4:22 (54.4)**	—	—
45–54 years old	—	19.9%,^***^ 4:31 (56.4)	—	—
55–64 years old	—	10.1%,^**^ 4:39 (57.9)	—	—
+65 years old	—	1.0%, 4:27 (55.6)	—	—
**Sex**
Women	100%, 2:51 (35.5)	69.1%,^***^ 4:17 (53.5)	—	—
Men	—	30.9%,^***^ 4:21 (54.2)	—	—

*Data for (A) Spanish version and (B) English version of the children's story are expressed as a percentage of users, mean duration of visualizations expressed as (minute: seconds), and as a percentage. In bold, the main contributors are indicated. The symbol “—” is meant for absence or data not reported or quantifiable, according to YouTube analytics. Statistics: Fisher exact test*,

** *p < 0.01;*

*** *p = 0.0001 vs. pre-pandemic period*.

**Table 3:** Comparative analysis on the use of the children's story on the cycle of life, death, and grief—geographic areas.

	**!Vuela Mariposa, Vuela!**		**Fly, butterfly, Fly!**	
	**(A) Historic**	**(B) Pandemic**	**(A) Historic**	**(B) Pandemic**
	**2009 to March 10, 2020**	**March 11 to August 31, 2020**	**2009 to March 10, 2020**	**March 11 to August 31, 2020**
**Geographical area**	***n* (%)**	***n* (%)**	***n* (%)**	***n* (%)**
Total visualizations	159,573 (100)	96,019 (100)	1,203 (100)	67 (100)
Mexico	**46,577 (29.2)**	3,644^**^ (3.8)	13 (1.0)	—
Spain	**14,268 (8.9)**	1,210^*^ (1.3)	10 (0.8)	—
Colombia	4,608 (2.9)	1,062 (1.1)	—	—
Argentina	3,049 (1.9)	569 (0.6)	—	—
Chile	1,354 (0.9)	312 (0.3)	—	—
Peru	359 (0.2)	302 (0.3)	—	—
**Ecuador**	272 (0.2)	81,524 (84.9)^**^	—	16 (23.9)^**^
USA	252 (0.2)	354 (0.4)	—	—
Costa Rica	219 (0.1)	—	—	—
Other Latino—American countries	^*a*^313 (0.1)	^*b*^48 (0.05)	—	—

*Data for (A) Spanish version and (B) English version of the children's story are expressed as real numbers (n) and percentages (%). In bold, the highest percentages are indicated. The symbol “—” is meant for absence or data not reported or quantifiable, according to YouTube analytics. Statistics: Fisher exact test*,

* *p < 0.05 and*

*** *p = 0.0001 vs. pre-pandemic period*.

a *Other Latino–American countries: Guatemala (95), Uruguay (62), Paraguay (41), Puerto Rico (37), Venezuela (35), Filipinas (20), El Salvador (12), Panamá (11)*.

b *Other Latino–American countries: Nicaragua (13), Bolivia (12), Panamá (12), El Salvador (11)*.

**Table 4:** Analytics of the viral video's engagement indicators.

	**Engagement's indicators**		
**Source**	**Visualizations**	**Time of visualization (h)**	**Mean duration of visualizations**
	***n*** **(%)**	***n*** **(%)**	***n*** **(%)**
**Total**	**23,246**	**2,069.1**	**5:20 (66.5)**
**Subscription status**			
Non-subscriber	23,161 (99.6)	2,064.7 (99.8)	5:20 (66.5)
Subscriber	85 (0.4)	4.3 (0.2)	3:04 (38.3)
**Type of device**			
Computer	16,439 (70.7)	1,628.7 (78.7)	5:56 (74.0)
Mobile pone	6,473 (27.9)	408.8 (19.8)	3.47 (47.2)
Tablet	161 (0.7)	13.1 (0.6)	4:52 (60.7)
TV	109 (0.5)	11.6 (0.6)	6:23 (79.6)
Video game console	1 (0.0)	0.1 (0.0)	8:01 (100)
**Traffic source**			
Direct or unknown	14,453 (62.2)	1,436.7 (69.4)	5:57 (74.2)
External sources	147 (28.5)	462.8 (22.4)	4:11 (52.1)
YouTube search	62 (6.5)	125.2 (12.0)	4:56 (61.5)
Other YouTube functions	36 (1.4)	24.2 (5.1)	4:24 (54.8)
Suggested videos	22 (1.1)	16.5 (3.9)	3:59 (49.8)
Exploration functions	11 (0.2)	1.9 (1.6)	2:39 (33.1)
Page of reproduction list	11 (0.1)	0.8 (2.0)	4:21 (54.2)
Reproduction lists	7 (0.0)	0.9 (1.1)	5:28 (68:2)
Channel's pages	1 (0.0)	0.0 (0)	0:27 (5.7)
**Geographic area**
Ecuador	22,903 (98.5)	2,047.5 (99.0)	5:21 (66.8)
United States	117 (0.5)	10.0 (0.5)	5:07 (63.7)
Mexico	42 (0.2)	1.8 (0.1)	2:34 (49.1)
Colombia	35 (0.2)	0.9 (0.1)	1.36 (42.6)
Spain	18 (0.1)	0.7 (0.0)	2:17 (32.1)
Chile	13 (0.1)	0.9 (0.0)	3:56 (28.5)
Peru	12 (0.1)	0.7 (0.0)	3:25 (20.0)
**Age**
13–17 years old	— (2.1)	— (25.9)	5:08 (63.9)
18–24 years old	— (11.1)	— (10.9)	4:59 (62.1)
25–34 years old	— (24.9)	— (24.7)	5:00 (62.3)
35–44 years old	— (25.5)	— (25.9)	5:08 (63.9)
45–54 years old	— (22.9)	— (23.0)	5:04 (63.2)
55–64 years old	— (12.3)	— (12.4)	5:95 (63.3)
+65 years old	— (1.2)	— (1.2)	5:01 (62.5)
**Sex**
Woman	— (69.7)	— (69.0)	5:01 (62.5)
Man	— (30.3)	— (31.0)	5:10 (64.4)

*Data for engagement indicators on the viral children's story are expressed as real numbers (n) and percentages (%). In bold, the highest percentages are indicated. The symbol “—” is meant for absence or data not reported or quantifiable, according to YouTube analytics*.

**Table 5:** Service used to share.

**Service**	**(A) Historic**	**(B) Pandemic**
	**2009 to March 10, 2020**	**March 11 to August 31, 2020**
	***n*** **(%)**	***n*** **(%)**
Total	3,944 (100)	3,005 (100)
WhatsApp	**1,958 (49.7)**	**1,803 (60.0)** *n.s*.
Copy to Clipboard	788 (20.0)	315 (11.1)
Facebook	708 (18.0)	334 (11.1)
Other services	207 (5.3)	437 (14.5)
Facebook Messenger	120 (3.0)	64 (2.1)
Gmail	69 (1.8)	27 (0.9)
E-mail	24 (0.6)	4 (0.1)
Google+	18 (0.5)	—
Text message	16 (0.4)	3 (0.1)
Twitter	12 (0.3)	6 (0.2)
Pinterest	12 (0.3)	3 (0.1)
Blogger	5 (0.1)	1 (0.0)
Hangouts	3 (0.1)	1 (0.0)
Reddit	1 (0.0)	7 (0.2)
Tuenti	1 (0.0)	—
LinkedIn	1 (0.0)	—
Embed	1 (0.0)	—

*Data for service used to share the viral children's story are expressed as real numbers (n) and percentages (%). In bold, the highest percentages are indicated. The symbol “—” is meant for absence or data not reported or quantifiable, according to YouTube analytics. Statistics: χ^2^ = 1.636, 1 df (degrees of freedom), p = 0.2008, n.s. (not statistically significant) vs. pre-pandemic period*.

**Table 6:** Content analysis of comments of users.

	**(A) Historic**		**(B) Pandemic**	
	**2009 to March 10, 2020**		**March 11 to August 31, 2020**	**Statistics**
	***n*** **(E:W:M) (%)**		***n*** **(E:W:M) (%)**	*P*
Participants	33 (4:26:3) (100)		57 (0: 44:13) (100)	
Grief	13 (0:11:2) (39.4)		5 (0:3:2) (8.8)	^***^
Personal loss	8 (0:6.2) (24.2)		1 (0:0:1) (1.8)	
Loss of a friend	2 (0:2:0) (6.0)		1 (0:0:1) (1.8)	
Loss at the school	3 (0:3:0) (9.0)		0	
Societal loss	0		2 (0:2:0) (3.5)	
Disenfranchised grief	0		1 (0:0:1) (1.8)	
Expressivity (use of emoji or emoticons)				
In grief	0		0	
Not in grief	0		11 (0:7:4) (21)	^**^
**Expressivity (number of words)**	***n*****, Mean** **±** **SEM**		***n*****, Mean** **±** **SEM**	
In grief	13, 37.5 ± 7.0		5, 17.0 ± 5.44	(^*^)
Not in grief	20, 9.8 ± 2.3	GGG	52, 16.1 ± 2.03	(^*^)

*Data for content analysis of comments of users on the viral children's story are expressed as real numbers (n); ratio of E, entities; W, women; M, men; and percentages (%). In bold, the highest percentages are indicated. The symbol “—” is meant for absence or data not reported or quantifiable, according to YouTube analytics. Statistics: Student t test of Fisher exact test; p < 0.001 vs. the comments not referring to grief;*

* *p < 0.05, one-tailed;*

** *p < 0.01;*

*** *p < 0.001 vs. the historic*.

### Instruments and Procedure

#### Ethnography—Analytics of the Children's Story Videos

All the data from the video publication date until August 31, 2020, were considered for the comparative analysis. As the two versions of the story's publication dates differ in 3 days, the publication date of June 25, 2009, was taken as a standard reference. It was verified that this did not affect the total computations. The data were obtained for three time intervals: the total and the two periods that comprise the historic or pre-pandemic period (from the beginning to March 10, 2020, inclusive) and the first 174 days of the COVID-19 pandemic (from March 11 to August 31, 2020). After that, the data of the viral version were analyzed on the day it went viral.

The virtual ethnographic analysis was made through three instruments. First, YouTube Analytics provides the traffic sources used to watch the video, geographical areas, sex and age of the users, and other social engagement indicators. The concepts behind the different variables are obvious or intuitive, but some may need to be defined. These are Traffic sources (the origin through which people found the site. YouTube traffic sources include search, browse features, playlists, and suggested videos, all power to varying degrees by the YouTube algorithm. Other sources include direct URL or external), viewing time (estimated total hours of viewing of the content by the audience), average view duration (estimated average minutes viewed per replay), impressions (number of times video thumbnails have been shown to viewers), and impressions CTR (click-through rate; views by impressions shown).

#### Ethnography of the Virality—Timeline, Engagement, Sharing, and Users' Feedback

The data to elaborate the two videos' comparative temporal maps were obtained through YouTube's analytical engine. Total dates were defined from June 25, 2009, to August 31, 2020. The temporal map of the evolution of COVID-19 for Ecuador was taken from the official source of COVID-19 alerts in Google, as offered as of September 1 (the latest to collect data possible for this publication) cites Wikipedia as a source, Creative Commons, free to use.

Analytics of the viral video's engagement indicators measured by the number of views, time of visualization, and the mean duration of visualization was obtained from YouTube Analytics for the day the video went viral. In addition, sharing was measured by the service used to share, for both periods, the historical and the pandemic.

Finally, for the users' feedback, content analysis on the comments posted by users in the viral video was performed. Based on the user profile, three participations or user's typologies were considered: E, entities; W, women; and M, men. Content analysis was done on users' comments to the video for both periods (historic and COVID-19). The presence of grief was recorded and qualified in five typologies as follows: personal loss (of a child, husband, father), loss of a friend, loss of a person in the school, societal loss (referred to COVID-19), and disenfranchised grief (loss of a pet). The content analysis searched for several items related to the video and the story, the emotions, and feelings raised; the cognitive aspects such as critical thoughts; a summary of the video and its interpretation, providing new opinions; consideration of its use; and the target population.

### Data Analysis

The data are presented and analyzed using quantitative and qualitative descriptive methods. Differences were analyzed with Student *t* test, χ^2^ test, or Fisher exact test. *p* < 0.05 value was considered statistically significant.

## Results

### Ethnography—Analytics of the Children's Story Videos

The video statistics provided by YouTube Studio for the video and its English version in the two periods of time, the pandemic and the history of the previous 11 years, are depicted in three tables according to three main analytical domains: (1) indicators and traffic sources (Table 1); (2) age and sex (Table 2); (3) geographical areas (Table 3).

#### Indicators and Traffic Sources

The database of indicators and traffic sources for “¡Vuela Mariposa!¡Vuela!” (Table 1) showed that from its publication in 2009 until August 31, 2020, a total of 255,592 visualizations and 336,980 impressions were recorded. The 37.6% (96,019) and 35.0% (118,088) of them, respectively, were done during the 174 days of the pandemic period recorded. 6,791.7 h, half of the total visualization time (13,454 h) was accumulated during the pandemic, which was as much as the 6,662.3 accumulated hours during the previous 11 years since its publication. The visualizations' mean duration was 4:14, a 52.8% of its total 8:01 duration, while the that of the previous years was 3:01, 37.6%.

During the preceding 11 years, the video captured 624 subscribers, 200 before the pandemic and 64.1% (400) of them in 174 days. The percentage of clicks during the pandemic was twice those of the previous years (10.0 vs. 5.5%). Compared to videos with the same duration, YouTube Analytics scored the audience's relative retention as “high” until minute 2:50 and “over the mean” until minute 6:05. The video received 892 likes vs. 51 dislikes and recorded a 94.6% active response to satisfaction, similar to the mean 94.4% given to the channel. The main traffic source changed during the pandemic with “director unknown” (48.6%) showing a statistically significant increase (χ^2^ = 30.570, 1 dg, *p* = 0.0001), as compared to historical, and leading the traffic, ahead of “external source” (34.1%) and “YouTube search” (12.2%), the main sources during the previous years (34.6 and 27.6%, respectively).

As a comparison, the indicators and traffic sources of the English version Fly, Butterfly! Fly! is indicated in Table 1. Since its publication in 2009, this version has obtained 1,270 total views and 641 total impressions, representing 0.05 and 0.19% of the original story's total activity, respectively. The 5.3% of total views were done during the pandemic, although views had a similar mean duration (1:27 vs. 1:39) and percentage of clicks (1.2 vs. 1.0) than the previous years. In contrast, the number of impressions was maintained relatively high, with 243 compared to 398 in previous years. Still, these indicators were small as compared to the viral video. From its publication, traffic sources were quite diversified, with 23.8% being “direct or unknown” and 23.8% “YouTube search,” followed by “suggested videos” (14.6%), “promotion of YouTube partners” (13.5%), or “external sources” (8.2%). During the pandemic, the “main traffic sources” also changed, as they were found concentrated on “suggested videos” (49.3%) and “direct or unknown” (38.8%) that significantly increased (χ^2^ = 25.023, 1 dg, *p* = 0.0001; 4.542, 1 dg, *p* = 0.0331, respectively) as compared to the historic.

#### Age and Sex

Since its publication, the age and sex of the audience have been very uniform, with 100% of visits being 35–44-year-old women, who spend a mean of 2:51 min in the visualizations, a 35.5% of the video duration. During the pandemic, the audience was broader, showing a normal distribution with the maximum in the 25–34 year (25.8%) and 35–44 year (24.8%) ranges, an increase that reached statistical significance except at the two tails of the curve (χ^2^s, age intervals from 18 to 54 years, *p* = 0.0001; 55–64 years old, *p* = 0.0015). The mean duration of visualizations per age group was similar, covering ~50% of the video duration, with the lowest time (3:46, 47.0%) in the youngest age group (13–17 years old) and the highest (4:39, 57.9%) in those 55–64 years old. Men also viewed the video, with a 30.9% participation, spending 4:21 min (54.2%) of visualization time than 69.1% of women, who spent 4:17 min (53.5%). No data were available for the English version.

#### Geographical Areas

The description of the geographical areas unveiled a key aspect since the video, which was popular among Mexicans (29.2%) and Spaniards (8.9%), became viral during the pandemic in another location, Ecuador (84.9%). The few data registered for the English version also pointed at Ecuador (23.9%) as a geographical area, whereas no information was available for the other areas.

### Ethnography of the Virality—Timeline, Engagement, Sharing, and User's Feedback

#### Timeline

**Figure 1:**
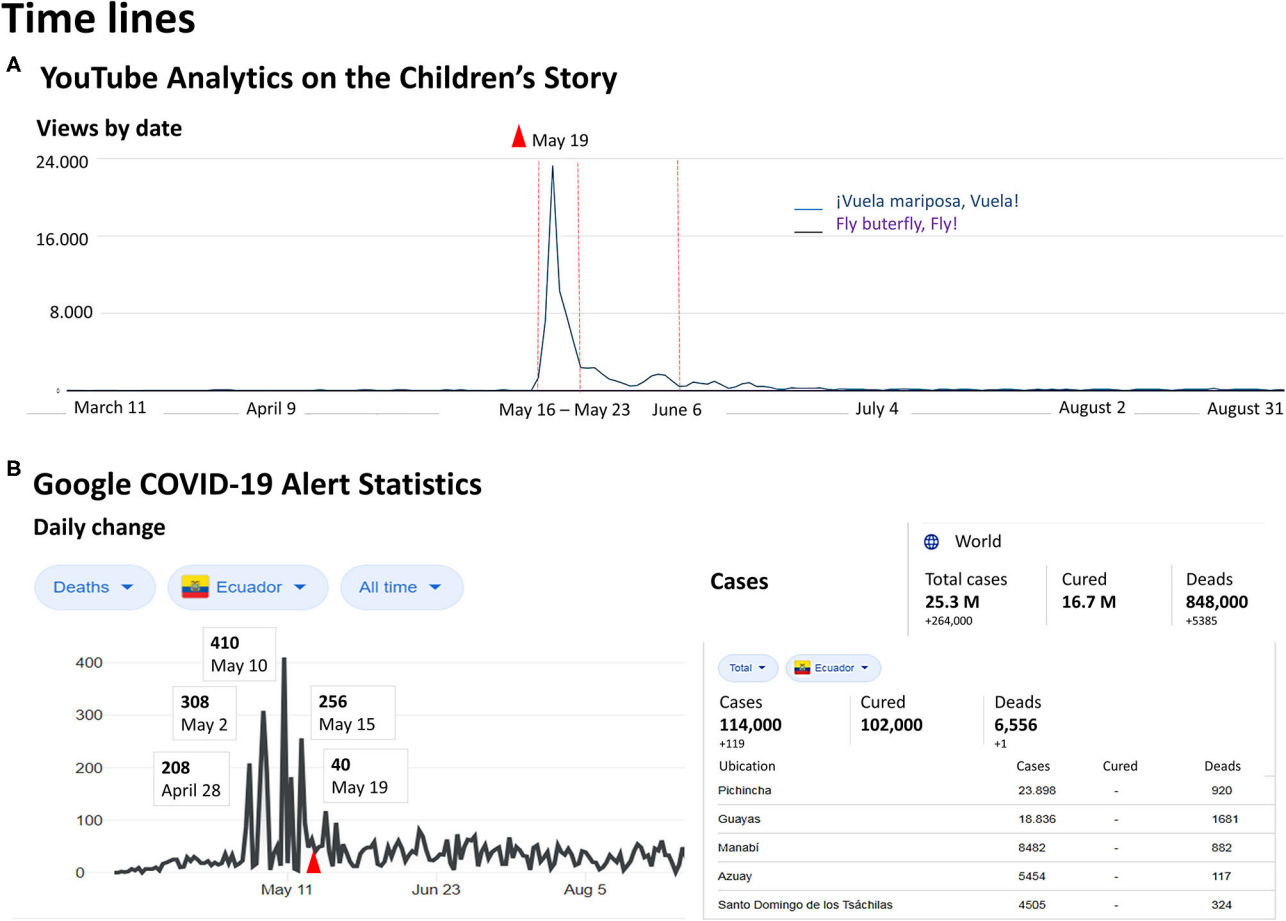
Time lines. (**A)** Temporal map of '1Vuela mariposa, Vuela! video's virality provided by YouTube analytics and views per day of the English version. **(B)** Synchronicity of the children's story virality (red icon) shown with Google COVID-19 alert statistics for Ecuador during the same period, March 11 and August 31, 2020. Right table: Total cases, cured and deads in the world, Ecuador and different Ecuadorian areas are depicted.

Figure 1 illustrates the temporal map of the video's virality provided by YouTube analytics and plots the views per day of the English version (hardly detectable) for comparison. During the pandemic, March 11 to August 31, 2020, YouTube referred to viral (+>999%) engagement, and May 19, 2020, as the day with a maximum of 23,246 visualizations and a retention time of 66.5% (5:20 min). Evident synchronicity is shown with Google COVID-19 alert statistics for Ecuador during the same period, March 11 and August 31, 2020. Total cases, cured, and deaths in the world, Ecuador, and different Ecuadorian areas are depicted.

#### Engagement Indicators

Viral video's engagement indicators (Table 4) show that 99.6% of views on May 19, 2020, were from non-subscribers, with a computer (70.7%) being the main type of device used to watch the video, and the mean duration was as high as 5:56 min (74.0%). Those who were already subscribers showed a lower 3:04 retention time. Access through mobile phones was 27.9%, and the mean duration was 3:47 (47.2%). Despite that tablets and TV were used in a hundred persons, the mean duration increased to 4:52 (60.7%) and 6:23 (79.6%), respectively. Only one user viewed the video from a video game console but saw it during its complete duration. Traffic source was direct (62.2%) followed by “External sources” (28.5%) with high visualization rates of 74.2% of video duration. Reproduction lists, while representing only seven visualizations, had a 68.2% retention time. Ecuador was the “main source” of the audience. Geographical areas also pointed at Ecuador as 98.5% of visitors with high retention times of 5:21 (66.8%). While the United States represented only 0.5% of the audience of that day, the retention was 5:07 (63.7%). The age and sex distribution replicated that described for the pandemic period, except for 13–17-year-old visitors who spent 63.9% (5:08 min).

#### Sharing

The “service used to share” analytics is depicted in Table 5. Since its publication, WhatsApp was the “main service” (49.78%) used to share the video, followed by “Copy to Clipboard” (20%) and Facebook (18.0%). During the pandemic, the sharing through WhatsApp was increased (60.0%) whereas the other two devices reduced their use to 11.1% in favor of “other services” (14.5%). The use of Facebook Messenger represented 3.0% of the shares since 2009, or 2.1% during the pandemic. Gmail, emails, Google+, text message, Twitter, Pinterest, Blogger, Hangouts, Reddit, Tuenti, LinkedIn, and Embed were minor.

#### User's Feedback as Commentaries

**Figure 2:**
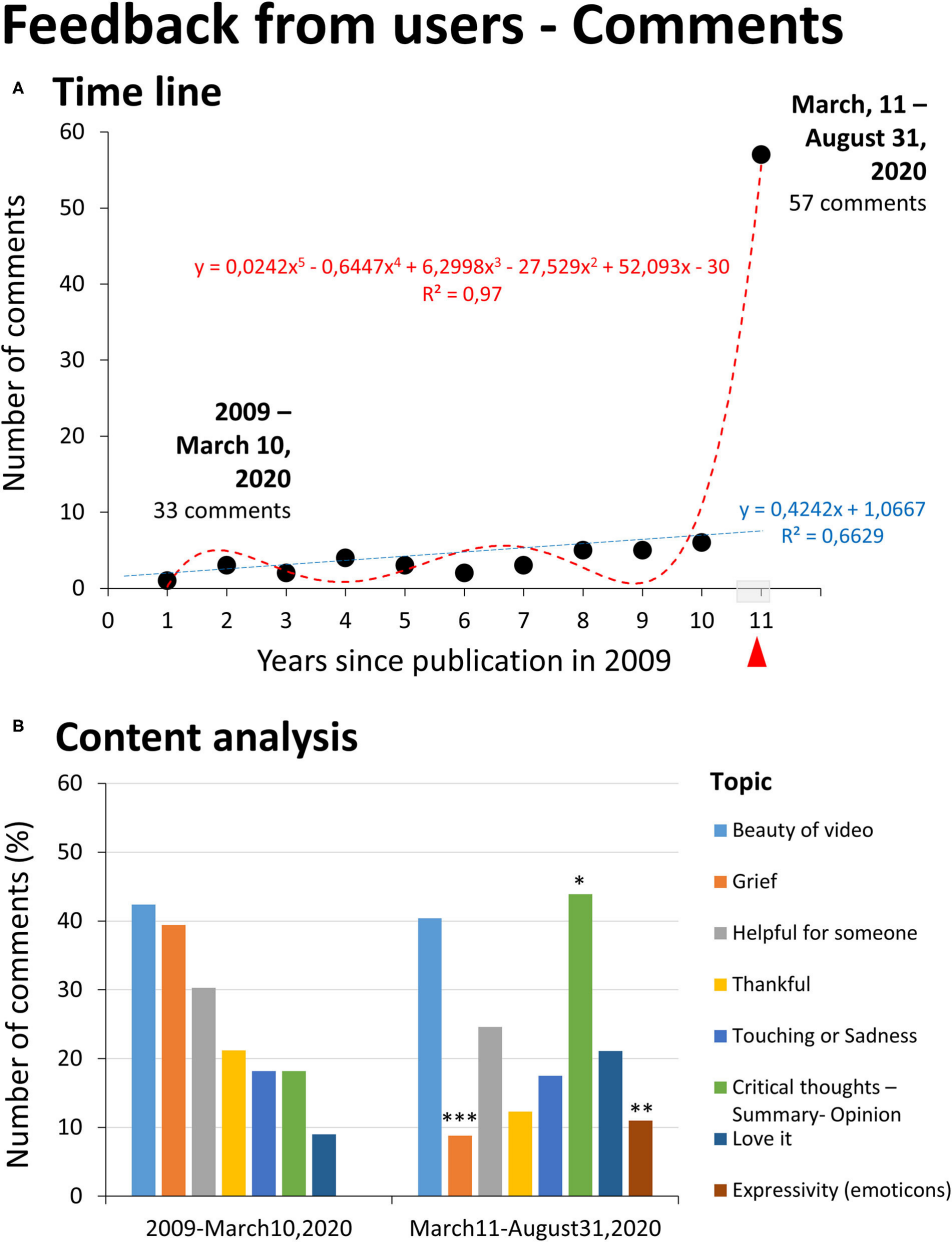
User's feedback as commentaries. **(A)** Time line, Statistics: Time line regression analysis. **(B)** Content analysis identifying 8 topics as described in the legend. Statistics: Chi-square, *p < 0.05; **p < 0.01; ***p < 0.001 vs. historic (2009-March 10, 2020).

Before the pandemic, the feedback from users recorded as commentaries to the video was low but increased with time, reaching a total number of 33 comments (Figure 2A). According to the user's profiles and written comments, the 33 comments were written by four bereavement counseling entities, 26 women and 3 men (Table 6). The exponential growth of comments was recorded during the 174 days of the pandemic studied, with 57 new commentaries of 44 women and 13 men. No entities contributed with comments.

The content analysis allowed us to identify eight topics (Figure 2B). During the years before the pandemic, the commentaries referred to the beauty of the video/story (42.4%) and/or described personal details about grief experiences (39.4%, 13 persons: 11 women, 2 men), primarily personal loss (24.2%) but also loss of friends (6.1%) or in the schools (9.1%). Those in grief wrote longer texts to express themselves (37.5 ± 7 words) than those not referring to grief, who used shorter texts (9.8 ± 2.3 words, Student *t* test, *p* = 0.0001). One-third of users (30.3%) found the video “helpful” for themselves or those who had lost a loved one, and they thanked for sharing (21.2%). Users qualified the video as touching or evoking their sadness (18.2%), whereas others referred to it as inviting to do reflections and/or expressed their opinion (18.2%). Thus, some users used the comments to summarize the story. Others extracted one or several messages regarding human qualities, existence, or values such as friendship, acceptance, and immortality of love. Other users used the commentary to provide their point of view and perspective. Finally, a few (9%) used or added an “I love it” to their commentary. This pattern was changed during the pandemic. The ratio of users referring to be in grief or evoking their grief during the semester of the pandemic was 5 (3 women, 2 men) over 57 participants (8.8%), whereas the previous averaged number per year was 1.18 ± 0.23 over 3.0 ± 0.47 participants per year (64.8%), 13 over 33 participants (39.4%) during the whole period (Fisher exact test, *p* = 0.0008). Societal loss, related to the current situation, was referred to in two of five comments. Evocation of the grief for the loss of a pet (considered disenfranchised grief) was also mentioned here for the first time. The other two explicit comments were for a personal loss and the loss of a friend. The length of comments with explicit reference to a grief experience was as long as those that did not (17.0 ± 5.44 vs. 16.1 ± 2.03) (vs. the previous years, one-tailed Student *t* test, in grief, *p* = 0.05; not in grief *p* = 0.04, respectively). Also, the use of emoticons was observed in the comments (Fisher exact test, *p* = 0.006). The content analysis confirmed the categorization of topics but changed their incidence. Thus, while the video's beauty was one of the two most common observations (40.4%), there was an increase in the number of users referring to critical thinking elicitation; users summarized the messages and some added opinions (43.9%). Some comments explicitly referred to watching the video as an assignment from their teacher or as docents, and these comments received positive reinforcement from others (up to 35 likes). The comments also qualified the video as helpful for their new role as bereavement counselors to help others.

## Discussion

The present study prompted by a children's story about the cycle of life, death, and grief hold in a YouTube channel for more than 11 years, suddenly becoming viral (+>999% increase of views) in May 2020, 3 months after the pandemic was declared (32). Therefore, because of its nature and singularity, this work is presented as a case study. To our knowledge, this report is the first original research that explores the ethnography of (i) a viral video, (ii) on death and grief taboo topics, (iii) for prescholars, and (iv) the change in the ethnography profile before and during the COVID-19 pandemic.

There is abundant literature about the role of social media and death and grief since the 1980s that emphasizes many of the arguments presented in the discussion of this work. New reports emerging in the advent of COVID-19 pandemic regarding crisis management, mental health challenges, and implications are also noteworthy (33). The original contribution of the present case study's analysis relies on the viral, exponential interest in this children's story in the specific scenario of COVID-19, presenting a different ethnographic profile compared to before the pandemic. The virality *per se* recognized a need for users to find and visualize material related to death and mourning processes that, as confirmed through the temporal overlapping with the tragic events in Ecuador and the user's feedback, mainly was aimed to help manage the sudden and dramatic number of deaths and consequent difficulties of the mourning process. The time when the video went viral was also determinant because time is considered a critical factor to help disrupt grief's vulnerability and support other protective factors (17). However, the most important finding was that the quantitative and qualitative ethnographic analysis allowed identifying a yet unknown severity of the pandemic situation in Ecuador that later could be confirmed by the timeline matches with the official records. The main port of Ecuador and the second most populated city, Guayaquil, was between April and May 2020, which experienced community contagion at an increasing rate. Reports referred to people suddenly collapsing and the deceased's bodies lying in the streets covered with white sheets because of the inability to be buried at once. In summer, Quito D.M., the country's capital, surpassed Guayaquil with the number of confirmed cases and became the new epicenter of the nation's pandemic (34). The geographic area analysis pointing to Ecuador as the video's virality's primary geographical location was also confirmed in the English version, in contrast to precedent Mexican and Spanish geographical locations during the historic. This indication is important since, despite the current devastating scenarios worldwide, the virality of a video on death and grief is a singular phenomenon in the context of the social and educational taboo of death. Here, it is interesting to note that during the previous years, Mexico, with a cultural syncretist tradition of indigenous rituals commemorating the dead and dead people, where monarch butterflies are a symbol of the deceased who are present on the Day of the Dead (4), was reported as the primary geographical location. Altogether, the geographic analysis was key for the ethnographic analysis, corroborated by the timeline and the users' feedback, unveiling the user's needs and use of the video to manage the mourning process, as will be later discussed.

In a formal comparative analysis, results obtained in a period are contrasted against a similar period immediately before or the same time frame in the previous year. In the present work, the idiosyncrasy of virality is significantly different from precedent data, at least for the number of visualizations. As the standard basal levels of the video were trimmed, the analysis was done compared to (1) the English version during the same period of the pandemic and (2) the historical records obtained during the previous 11 years.

The indicators showed that virality allowed to achieve, in a brief time, approximately half of the amount of activity accumulated during the precedent years, as measured by the number of visualizations and impressions. Viewing times of virtual social media are usually very short and, in most cases, rarely exceed 3 min. Therefore, the average viewing duration of the video was greater than the standard and may support its educational use/purpose. Besides, the fact that the time of visualization was equal to pre-pandemic and the mean duration of the visualizations was longer talks in favor of the video's potential impact at the educational level. It also helps to discard that the virality resulted in a sudden but short exposure to the video content, which would reduce this potential. The 2-fold increase in the number of subscribers and the percentage of clicks, which are relevant to measure the impact and quality of the materials, suggests that the viral video was translated into the channel's adherence. Here it is important to note that, as a comparison, these variables were not modified in the English version. It would have been interesting to compare the profiles with those of another contemporary children's story addressing other physical changes or loss in the natural life cycle, such as the first tooth loss. Other interesting comparisons could be made with virtual material about fear of death when experiencing natural disasters. Recent reports on higher acute grief after death due to COVID-19 compared to natural loss allow the researchers to predict that pandemic-related increases in pathological grief are foreseen as a worldwide public health concern (35).

Traffic sources were also informative about the way the video went viral. During the pandemic, the access to the video was direct, in contrast to external sources or YouTube search as main sources used during the previous years. Incoming traffic channeled through websites or direct recommendations via links means that other people participate in the election. These indicators suggest a goal-directed behavior of users compatible with educational purposes. The posterior analysis of sharing was also illustrative and confirmed that WhatsApp's direct links were the most used service to spread the word.

Apart from the geographical area, age, and sex were two relevant socio-demographic factors to identify the audience's profile, which expanded from all cases being “35–44 year-old women” user profile to a broader normal distribution of ages, also including the presence of men. Age and sex factors are critical to determine the users' profile and elucidate the potential needs of users covered by the material. Thus, during the historic, the adult female profile would respond to mothers confronting the grief process or women in parenting or professional roles toward pre-scholars, as confirmed in the content analysis of the user's feedback. Similarly, the broader coverage of ages and sexes during the pandemic was informative of new use of the pre-scholar material among teenagers as academic assignments or men as fathers/docents. This information helped define subsequent actions for the audience, as a target population, in agreement with other efforts to elaborate and share action lines for death in preschool education (14). Thus, a YouTube video call in the channel offered a guide for education on death and mourning for adults (parents and docents) and organized a webinar on this topic. The resulting survey's ethnographic analysis allowed defining potential professional users' sociodemographic and socioecologic profiles (20). These data were valuable to address their needs on this topic in an immediate and personalized manner. Still, in future lines of analysis and study, within the most rigorous field of education through the use of audiovisual elements, it would be interesting to qualify the video using DISCERNMENT score, a content reliability index, or PEMAT, another evaluation instruments of educational materials for patients for audiovisual materials (8). The level of comprehensibility and the capacity for action the video may allow could be assessed using these scales.

The temporal maps showed that the video became viral immediately after the most severe days of the pandemic outbreak in Ecuador. Among the engagement indicators on the day the video went viral, it is noteworthy that non-subscription as the majoritarian status, the direct traffic source indicating direct connection, and the use of a computer as a device instead of mobile phones, despite WhatsApp was identified as the service used for sharing using the Share on YouTube button. The overrepresentation of females in the two studied periods is common and agrees with bereavement research using voluntary-response sampling and is considered that it may reflect a more vital need for women to share their feelings (19). In this respect, therefore, in the present work, the emergence of masculine participation in the users' profiles and the users' feedback is noteworthy.

The user's feedback to the video can be considered an important exercise to deal with the “word taboo” (verbal omissions related to a taboo topic) (1–3). Despite the low translation of the video virality into written comments, the exponential increase shown in the timeline was noticeable. This is important because the taboo of death also extends to “words taboo” and strongly contributes to grief stigmatization and disenfranchised grief symptoms. Also, although death rituals and mourning practices are highly dependent on the cultural context (5, 36), the COVID-19 pandemic has created a global scenario with commonalities for people in all nations [i.e., (19, 37–42)]. The most important commonalities are the psychological burden associated with confinement (43), the inability to say goodbye or to perform rituals according to believes and culture, and the measures of physical distancing, all of them considered risk factors for traumatic and disenfranchised grief in people with low resistance or resilience (19, 35, 44, 45). Thematic content analysis of Twitter data from bereaved family members and friends (46) or national newspapers (28) has also reported the complexity and difficulty of the current bereavement scenarios.

In the present work, the comparative content analysis highlighted a change in the user's profile concerning the mourning process. This aspect is important as listening and understanding the user's opinions must improve the cultural and pedagogic quality of death education (47). In the precedent years since the publication of the children's story, users referred to the personal loss of a child, another family member, or a student wrote narrative comments to explain the vital crisis they were confronting and expressed their depth sorrow. In addition, some comments were provided by entities specialized in grief management, providing support to bereaved families. In this latter case, users' written comments giving spontaneous feedback to the video could be understood as a kind of virtual grief group where bereaved people amenably expressed their loss personally and socially.

The content analysis of the comments written during the pandemic indicated societal mourning and included disenfranchised grief. The broader range of age of users, including the youth, would also explain the use of emoji and emoticons to express emotions among those not in grief. However, the most relevant difference was that, in agreement with the current situation, most of the comments reflected critical thinking and provided a space for users to summarize the meaning of the children's story. They were also used to provide their own opinion on the scale of values and other important issues related to the cycle of life, death, and mourning. In this sense, this change of expressivity patterns suggests that the material covered the personal needs of bereaved mothers/professionals during the historic, whereas the educational purpose and use of the video were more evident in the new context of COVID-19. As mentioned in some comments, the video was promoted from educative scenarios as an academic assignment for teenagers. Similar to grief support being part of health education (48), the educative areas also showed a strong need to train their fellows. Besides, the commentary forum worked as a scenario to establish symbolic family bonds among unknown people worldwide, similarly to what has been recently described in the organizational ethnography of charities in crisis times (49). In addition, recent grief research using voluntary recruitment before and during the pandemic showed no significant differences in socio-demographic and loss-related variables (19). However, in the present work, a singular change in the ethnography profile before and during the outbreak was determined by viewers' spontaneous and voluntary enrolment.

Understanding the nature and pattern of misinformation infodemic during large-scale disease outbreaks deserves special attention (50). When referring to social media as a source and resource of health information, the topic is controversial, under constant evaluation and debate. This is primarily due to the worrisome number of videos found to present medically misleading information and some patients' abusive behavior using these resources (8, 51). In recent medical online education reports, social media as a medical information source during the COVID-19 pandemic is critically analyzed (6). Furthermore, the infodemics of the COVID-19 pandemic are also found among healthcare students and professionals (52). Despite these aspects, the analysis of engagement on social media networks and digital newspapers shows that good practices may find a promising scenario for the new native digital generations (46, 53, 54) and can be even foreseen as a palliative social media (55). The present report supports the emerging studies in this pandemic, showing that goal-directed social networks' engagement in health media and healthcare professionals plays an important role (6, 8, 51, 56).

The most important limitation to this work is intrinsic to the virality nature of the material and that the ethnographic analysis focused on a case study. Besides, the singularity video's virality associated with a specific scenario could also be considered a limitation. Other limitations include those related to the sources provided by the analytics of this social media, the spontaneous and voluntary-response sampling, and self-reports in the user's feedback.

In summary, this ethnographic analysis on a case study provided evidence that, under singular circumstances, a YouTube video dealing with the idea of death, a taboo topic even in its most censored forms such as disenfranchised grief presented in the children's story, was able to go viral. The quantitative and qualitative analyses identified a change in the users' profiles, engagement, and feedback. The analysis pointed to Ecuador as the geographical niche of the viral virus before the severity of the nation's pandemic scenarios was known. Engagement by non-subscribers, direct traffic sources, and mean visualization times suggested educational purposes as confirmed by the users' feedback enriched by critical thinking, referring to the cycle of life's meaning and societal mourning. The broad coverage of all age ranges and the inclusion of male gender talk favoring this virtual resource's potential and flexibility allowed an immediate switch of users' profiles responding to their vital crisis needs. Thus, the ethnography pointed at YouTube as a flexible education resource, immediately reaching diverse users and being highly sensitive to critical events. Good practices on using YouTube as a source and resource of health education can make it a promising tool for native-digital users and precedent generations. More importantly, it talks in favor of good practices in this popular social media as eligible as “palliative social media,” helping to mitigate the death taboo in the current Western societies in a world devastated by the COVID-19 pandemic.

## Data Availability Statement

The original contributions presented in the study are included in the article/Supplementary Material, further inquiries can be directed to the corresponding authors.

## Ethics Statement

Ethical review and approval was not required for the study on human participants in accordance with the local legislation and institutional requirements. Written informed consent for participation was not required for this study in accordance with the national legislation and the institutional requirements.

## Author Contributions

The present research is the result of the work of LG-L.

## Conflict of Interest

The author declares that the research was conducted in the absence of any commercial or financial relationships that could be construed as a potential conflict of interest.
